# Study of Factors Influencing Dry Eye in Rheumatoid Arthritis

**DOI:** 10.1155/2020/5768679

**Published:** 2020-08-08

**Authors:** Weifang Ma, Guoliang Wang, Xiaofeng Li, Huping Wu, Zuguo Liu, Nuo Dong, Cheng Li

**Affiliations:** ^1^Eye Institute & Affiliated Xiamen Eye Center, School of Medicine, Xiamen University, Xiamen, China; ^2^Fujian Provincial Key Laboratory of Ophthalmology and Visual Science, Xiamen, China; ^3^Department of Ophthalmology, Affiliated People's Hospital & Zhenjiang Kangfu Eye Hospital, Jiangsu University, Zhenjiang, Jiangsu, China

## Abstract

**Purpose:**

The present study investigated the relationship between dry eye and the disease activity in patients with rheumatoid arthritis (RA).

**Methods:**

Patients with RA were divided by the Ocular Surface Disease Index (OSDI) into the symptomatic group (score ≥ 12) and the asymptomatic group (score < 12). By using the Disease Activity Score (DAS-28) questionnaire, they were divided into the active group (score > 2.6) and the stable group (score ≤ 2.6). In the control group, 20 healthy adults with matched sex and age were selected. RA patients and healthy adults were inspected for the tear film break time (TBUT), tear meniscus height (TMH), corneal fluorescein staining (CFS), meibomian scan (MS), meibomian gland secretion score (MSS), and eyelid margin assessment (EMS).

**Results:**

The TBUT of the RA group was significantly less than that of the control group, while the CFS, MS, EMS, and MSS were higher. The TBUT of the symptomatic RA group was significantly less than that of the asymptomatic group, and the CFS was higher. In the active RA group, only the CFS was higher than that of the stable group, and there was no significant difference between the two groups for other parameters. Furthermore, there was no significant correlation between the course of RA and the dry eye (*P* > 0.05).

**Conclusion:**

The rheumatoid activity does not necessarily lead to an aggravation of dry eye. Regardless of the duration, RA was not found to exhibit relation with the severity of dry eye. *Translational Relevance*. RA patients with disease active period cannot be ignored for the existence of dry eye, since patients with dry eye often lack the signs and symptoms.

## 1. Introduction

Rheumatoid arthritis (RA) is a chronic systemic autoimmune disease and characterized by joint synovitis and multiple symmetric small and large joint lesions. It is the most commonly observed type of inflammatory arthritis. The incidence of RA in the world and China is about 0.02% and 0.017%, respectively, and the prevalence is about 0.5–1% and 0.35–0.42%, respectively [1–5]. However, in some specific groups, such as the Pima Indians in North America, the incidence can be as high as 2-2.1% [6]. The proportion of women in RA patients is about 60–75%, which is 2-3 times of men, and the disease occurs between fourth and sixth decades of life [6, 7]. In general, the prevalence of RA goes on increasing with age; however, there is no sufficient evidence to suggest that the incidence changes with age. The severity of RA with age, disease activity, and extra-articular manifestations has decreased, which may be related to new treatments, drugs, and other factors [8–10]. In addition, the mortality of RA tends to decline over time [11] but still is higher compared with the general population [8].

RA is mainly considered as a joint disease, but it has the capacity to inflict multiple organs because of the abnormal systemic immune response. About 17.8–47.5% of patients present the extra-articular manifestations [12, 13], among which the ocular surface manifestation is the most common [14, 15], which seriously affects the quality of life [16]. Earlier studies have shown that synovitis is the pathological basis for joint injury in RA [5]. Due to the complex interactions between the genes and environment, the immune tolerance is destroyed and synovitis gets presented in a typical symmetrical pattern [17]. In some cases, the rheumatoid factor (RF)—a group of autoantibodies that activate the complement pathways—can cause the extra-articular damage [18]. Most of these complications are pathologically based on pannus [5] and may occur in the skin, eyes, lungs, heart, kidneys, blood vessels, salivary glands, central and peripheral nervous systems, and bone marrow. The high mortality in RA is mainly associated with the extra-articular complications [12, 13, 18].

It has been reported that the incidence of dry eye in the general population is about 5–17% [19–22], while in the patients with RA, it is as high as 19–31% [23–28]. Recent studies show that the pathogenesis of dry eyes is similar to the other extra-articular complications of RA, which may be a mucosal autoimmune disease [29, 30]. It is speculated that the degree of dry eye may also change with the systemic conditions, due to the influence of systemic immune response. Therefore, this study intended to explore the following questions through the detection indicators of dry eyes in RA patients: (1) the relationship between dry eye and disease activity in patients with RA; (2) the difference in the extent of dry eye in RA patients with and without dry eye symptoms; (3) to find the correlation between the degree of dry eye and the course of disease.

## 2. Methods

### 2.1. Patients

A total of 30 adult RA patients (including 6 males and 24 females) were recruited from the Xiamen Eye Center, affiliated to the Xiamen University, between October 2016 and December 2018, which were selected on the following basis: (1) patients who came to the hospital for medical treatment; (2) community screening for cataract; (3) social recruitment using the Internet. All of the patients were previously diagnosed with positive or typical RA, thereby fulfilling at least 4 of 7 criteria as required by the American College of Rheumatology in 1988 [31].

Patients with previously known allergic conjunctivitis, any corneal lesions, chronic dacryocystitis, trachoma, blepharitis and other ocular surface diseases or primary or secondary Sjögren's syndrome, hyperthyroidism, diabetes, and other systemic diseases that can affect the tear secretion and tear film stability, were excluded. Also, the patients who had undergone an ocular surgery or who used worn contact lenses or any eye drops or visual display terminal for more than 4 hours per day were excluded.

Twenty healthy adults (including 4 males and 16 females) with matched sex and age were selected as the control group that included the cataract patients before surgery and the hospital staff.

### 2.2. Questionnaire

All the RA patients were graded by the Ocular Surface Disease Index (OSDI) questionnaire, a reliable and commonly used survey in the assessment of symptoms of ocular surface disease [32]. It consisted of 12 items that assessed the symptoms, functional limitation, and environmental factors related to dry eye [33], with an overall score of 0–100. The severity of ocular surface diseases assessed by OSDI was graded as follows: 0–12: normal; 13–22: mild; 23–32: moderate; 33–100: heavy [34]. Another survey used to assess the rheumatoid arthritis was the disease activity questionnaire (DAS-28) [35], which evaluated the number of tender and swollen joints (28-joints count), self-assessment of disease activity of the patients, and erythrocyte sedimentation rate (ESR) [36]. According to the OSDI, the patients were graded into the symptomatic group (score ≥ 12) and the asymptomatic group (score < 12). According to the DAS-28 scores, they were graded into the active group (score > 2.6) and the stable group (score ≤ 2.6).

### 2.3. Measurement of Tear Film Break Time (TBUT)

The ocular surface analyzer (Oculus keratography: SIRIUS-ANTARES (Italy C. S. O. SRL)) was used in the inspection of TBUT. The procedure involved is as follows: patients who were able to cooperate were asked to blink their eyes twice after the prior adjustment of the focal length, and then keep open, until the eyes automatically blinked or till the sampling procedure ended. Patients who faced difficulty to cooperate were asked to keep their eyes open with silent counting for 10 s at the beginning of the examination. The obtained test results were recorded for all the patients.

### 2.4. Measurement of Tear Meniscus Height (TMH)

The ocular surface analyzer (Oculus keratography: SIRIUS-ANTARES (Italy C. S. O. SRL)) was used in the inspection of TMH. The procedure involved is as follows. The lower tear meniscus was focused and the hand putter was adjusted to make the focus clear. The acquisition button was quickly clicked to take the images. If the lacrimal stream of the patient was narrow and clear images could not be obtained under infrared light, the white light source was selected for collection. The ruler button on the interface was selected and pulled from the upper edge of the tear river to the lower edge for measuring the tear meniscus height. This value gave the height value of the tear river.

### 2.5. Meibomian Scan (MS)

The ocular surface analyzer (Oculus keratography: SIRIUS-ANTARES (Italy C. S. O. SRL)) was used in this inspection. The procedure involved is as follows. The upper and lower palpebral conjunctiva was, respectively, exposed to the infrared meibomian gland imaging system to get a clear image of the morphology. The image of white stripes showed the meibomian gland, and the degree of lack of glands was scored as follows [37]:

  0 = no loss (normal)
  1 = gland deletion <1/3 of total area (mild)
  2 = loss 1/3 to 2/3 (moderate)
  3 = more than 2/3 loss (severe); the scores of upper and lower eyelids were added and scaled in the range 0–6

### 2.6. Corneal Fluorescein Staining (CFS)

The slit-lamp microscope (SL990N (Italy C. S. O. SRL)) was employed in this inspection. A fluorescein paper was moistened with normal saline and subjected at the lower fornix of the patient. Patients were asked to close and open eyes for the proper distribution around the cornea. The number of dot stains obtained were observed under the cobalt blue light, and scores were assigned as follows: 0 = no stain; 1 point = 1∼10 dot staining; 2 points = 10∼30 dot staining; 3 points = point stain fusion, filaments, and ulcers.

### 2.7. Measurement of Meibomian Gland Secretion Score (MSS)

After hot compression for 10 minutes, a digital pressure was applied to the tarsal plate by two cotton buds to squeeze the meibomian secretions, and the quality of the expressed lipid was observed and graded as follows [38]: 0 = clear fluid; 1 = cloudy fluid; 2 = cloudy particulate fluid; 3 = inspissated (like toothpaste).

### 2.8. Measurement of Eyelid Margin Assessment (EMS)

A Slit-lamp microscope (SL990N (Italy C. S. O. SRL)) was used for this study. The upper and lower eyelids were gently turned to expose the eyelid margin, and the following conditions were observed for their presence or absence: (1) the eyelid margin was hyperemic and round; (2) irregular eyelid margin; (3) blocked meibomian gland openings; (4) the skin mucosal boundary (Marx line) moved forward or backward. Scoring rules were as follows: 1 point or presence of each condition, 4 points in total; 0 for absence of all.

### 2.9. Statistical Analysis

Statistical analysis was performed using the SPSS 23.0 software; descriptive statistics methods were used for baseline characteristics (Mean ± SD). The Mann–Whitney *U* test was used to compare the discrete variables, and Spearman correlation test was computed to assess the dry eye and RA duration. The statistical significance was considered to be *P* < 0.05, and results were given with their 95% CIs.

## 3. Results

**Figure 1:**
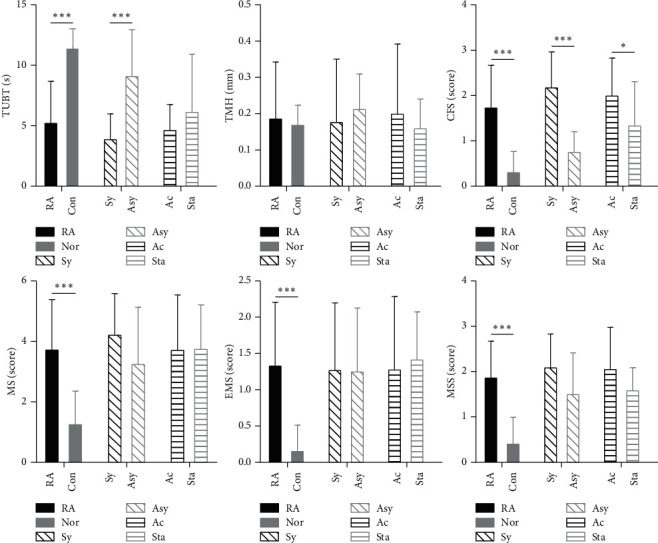
The Mann–whitney *U* test of every examined items in RA patients and control group (RA and Con), symptomatic group and asymptomatic group (sy and asy), and active group and stable group (act and sta). (^*∗∗∗*^*P* < 0.001, ^*∗∗*^*P* < 0.01, and ^*∗*^*P* < 0.05).

The control group (Con) and RA patients (RA): there were no significant differences in the age and sex between the two groups. TBUT of both the groups was significantly different and was significantly lower for the RA group (*P* = 0.001). The scores of MS, CFS, MSS, and EMS were significantly higher in the RA group than those of the normal (*P* ≤ 0.001), while there was no significant difference between both the groups for TMH (*P* = 0.331) (Figure 1; Table 1).

Symptomatic group (Sy) and asymptomatic group (Asy): according to the OSDI, there were 8 patients in the asymptomatic group and 22 patients in the symptomatic group, with no significant differences in the age and gender. Furthermore, there were no significant differences between the two groups in the TMH, MS, MSS, and EMS parameters (*P* > 0.05). The TBUT of the two groups was significantly different, and for the symptomatic group, it was significantly lower (*P*=0.002). The CFS score was higher in the symptomatic RA group than in the asymptomatic RA group, and the difference was statistically significant (*P*=0.001) (Figure 1).

Active group (Act) and stable group (Sta): A total of 18 patients in the active RA group and 12 patients in the stable RA group were found after evaluation using the DAS-28 questionnaire. There were no significant differences in the age and gender between the two groups. The CFS scoring showed a significant difference between the two groups, and for active group, it was significantly higher (*P*=0.040) (Figure 1).

**Figure 2:**
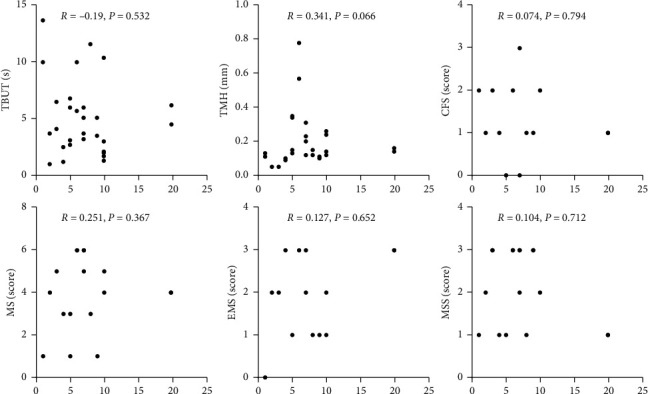
Correlation between the duration of RA patients and each ocular tests. The *P* value was calculated by the spearman correlation test.

Duration (Dur) and dry eye (DE): the Spearman correlation test showed there was no correlation between the duration of the disease and the dry eye test items (*P* < 0.05) (Figure 2).

## 4. Discussion

The present study was aimed to explore the relationship between dry eye and the disease activity in patients with RA. The results of our study suggest that the patients with RA exhibited a shorter tear film breakup time, severe meibomian gland loss, degenerated blepharon lipids, severe corneal epithelial injury, and obvious changes in the blepharon morphology compared with the control group. Interestingly, there was no significant difference in the tear meniscus height between the two groups. The tear meniscus is an integral part of the tear film and the “repository” of tears. The amount of tears in the tear meniscus is about 75–90% of the total [39]. Although the measurement of tear meniscus height is one of the important indicators of the dry eye examination, it can merely reflect the level of tear secretion at that time. The mandatory open pattern required by the commonly used noninvasive ocular surface comprehensive analyzer may affect the measurement of lacrimal height due to the secretion of reflex tearing. Studies have found this condition even in the patients with aqueous tear deficiency [40]. Furthermore, in the patients with subjective dry eye symptoms, the reflex tear secretion was close to normal. The possible reasons for such results are speculated as follows: (1) RA patients are in the state of reflex tear secretion for a long time; hence, the tear secretion is maintained in the normal range; (2) RA patients may not have a reduced tear secretion, and the discomfort of subjective dry eye may be caused by other reasons.

The RA group exhibited higher CFS scores than those of the control group, and the dry eye symptoms group showed higher CFS scores than those of the asymptomatic group, with statistically significant differences. Lam et al. [41] reported that the level of cytokine interleukin—17 (IL—17) in tear was associated with the severity of the CFS. Lee et al. [42] showed that the corneal damage was caused by the excessive expression of proinflammatory cytokines of tear, and the epithelial damage due to the presence of cytokines of cell damage was severe than that due to the lack of water. In the RA patients with dry eye, the more heavily stained cornea indicated a more severe condition of the dry eye. Inflammatory cytokines may play an important role in the development of dry eye. Some scholars believe that, as the cornea is close to the palpebral conjunctiva and easily exposed to inflammatory cytokines expressed in the vessels, there is an increased chance of injury. However, the relationship between the tear components, including cytokines and dry eye, needs to be further explored.

There were significant differences between the RA group and the control group for TBUT and the meibomian gland examination. This suggests that the patients with RA may have varying degrees of meibomian gland dysfunction (MGD). The meibomian gland is the largest sebaceous gland in human body, and its main function is to secrete sebum and to form the outermost structure of tear film. The meibomian gland plays an important role in maintaining the stability of tear film. TBUT is currently a reliable indicator for assessing the tear film stability. Our results suggest that the TBUT of RA patients was shorter than that of the normal group and was shorter in the symptomatic RA group than that of the asymptomatic RA group. Thus, it can be concluded that the tear film stability is worse in RA patients, especially those with dry eye symptoms. It is speculated that the reason for poor tear film stability in RA patients may be caused by the abnormalities in the meibomian gland. There are many causes of MGD [43], which may be due to the attack of the immune system on one of the target organs, as in the case of Sjögren's syndrome [44].

The quality of tear film is closely related to the lipid layer formed by the sebum secreted by meibomian gland. Poor quality of the lipid layer not only affects the formation of tear film but also causes excessive evaporation of tears. Cornea protected by the tear film can be affected indirectly. Our results suggest that patients in the symptomatic group exhibited higher CFS scores, which means more spots were evident on the corneal epithelium. It was speculated that patients in the symptomatic group showed a more severe corneal damage.

The disease assessment of RA can not only compare the effects of treatment at different stages of individuals but also help to assess the progress of disease. According to the clinical observations, we expected that the disease activity might be positively correlated with dry eye and patients in the disease active stage might have more severe dry eye. However, our results suggested that there was no much significant difference between the active or stable groups, except for the CFS. We assumed that in the beginning of the active stage in RA, the patients may have more serious dry eye condition. However, our experimental results (Figure 1) suggested that the patients in stable stage of RA showed similar severity of dry eye to those in active group. Detailed dry eye examinations should be paid to all the RA patients, as patients in the stable stage exhibited mild dry eye. In addition, these results were consistent with the study of Miho Fujita et al. on the RA patients with nonsecondary Sjögren's syndrome [45]. Schargus et al. [46] found that the disease activity and tear osmotic pressure showed a positive correlation.

TBUT and TMH were used to evaluate the quantity and quality of the tears. Our results indicated that the RA disease activity could be possibly related to the secretion of tears and no obvious correlation existed for the tear stability, although the disease activity can still have an influence on the tear composition.

Our analysis of the disease course of RA and dry eye suggested that no significant correlation existed between them, although studies have found that patients with RA disease activity for long duration can influence all aspects of the quality of life [47]. Since the dry eye is a multifactor disease and has certain compensation, the influencing factors of dry eye may not be RA disease itself. Hence, influenced by age, gender, and other factors, even in short duration of disease, the degree of dry eye may be more severe.

To sum up, we found that patients with RA can have a meibomian gland dysfunction (MGD), but the functional and morphological differences with simple MGD need to be verified by further experiments. Besides, most studies showed that the dry eye in RA patients was due to the secondary Sjögren's syndrome, but our findings suggest that RA patients (without secondary Sjögren's syndrome) did not exhibit poor tear secretion. However, we cannot rule out the influence of the tear reflex, and further evaluation of lacrimal gland function needs to be understood.

Our study proposed the following three aspects. First, the RA disease activity may not necessarily worsen the dry eye symptoms. On the contrary, the patients in the period of the active disease cannot be ignored for the presence of dry eye, since patients with dry eye often lack the signs and symptoms. Hence, irrespective of the progression of the RA disease activity, the possibility of dry eye should be evaluated and clinical intervention can be taken if necessary. Secondly, when patients with RA complain about dry eye symptoms, it may indicate the damage to the corneal epithelium. For such patients, a better attention needs to be paid to the corneal epithelium, which should be carefully examined and dealt with in time. Finally, the duration of RA may exhibit no correlation with the severity of the dry eye. Patients with RA should get checked for the presence of dry eye, regardless of the duration of the disease. The evaluation of the dry eye should be based on the clinical manifestations and symptoms, combined with the objective examination indexes.

The advantages of this study lie in the consistency of the clinical methods and prospective recruitment of the RA patients. Clinical examinations were evaluated by a same physician in a standardized way. There were few shortcomings in this study. (1) This was a small sample study, which may face limitations in reflecting the actual situation. An increased sample size can prevent such shortcomings. (2) The examination and evaluation of dry eye were not comprehensive. For example, the composition changes of tear and the morphological and functional examination of meibomian gland were insufficient. (3) This study failed to consider whether the treatment drugs for RA such as hormones and immune-suppressants exhibited any effects on the dry eye. This deficiency can be minimized by making a comparison between the patients treated with such drugs and those not.

## Figures and Tables

**Table 1:** Dry eye assessment and statistical results between each groups.

	RA	Con	*P*	Ac	Sta	*P*	Sy	Asy	*P*	*R*	*P* (Dur and RA)
Mean age	51.933 ± 9.89	50.150 ± 9.433	0.463	49.400 ± 10.762	57.000 ± 5.249	0.055	50.454 ± 10.220	53.500 ± 7.270	0.730		
Male/female	6/24	4/16	0.533	4/14	2/10	0.397	4/18	2/6	0.534		
TBUT (s)	5.267 ± 3.478	10.868 ± 1.816	0.001	4.545 ± 2.066	6.710 ± 5.147	0.619	3.918 ± 2.128	8.975 ± 3.886	0.002	−0.119	0.532
TMH (mm)	0.186 ± 0.158	0.150 ± 0.052	0.331	0.211 ± 0.184	0.137 ± 0.069	0.619	0.177 ± 0.175	0.213 ± 0.099	0.097	0.341	0.066
MS	3.733 ± 1.710	1.250 ± 1.118	0.001	3.800 ± 1.814	3.600 ± 1.673	0.746	4.182 ± 1.471	2.500 ± 1.915	0.156	0.251	0.367
MSS	1.867 ± 0.834	0.400 ± 0.598	0.001	2.000 ± 0.943	1.600 ± 0.548	0.307	2.091 ± 0.831	1.250 ± 0.500	0.097	0.104	0.712
EMS	1.333 ± 0.900	0.300 ± 0.470	0.001	1.200 ± 1.033	1.600 ± 0.548	0.267	1.455 ± 0.934	1.250 ± 0.957	1.000	0.127	0.652
CFS	1.733 ± 0.961	0.150 ± 0.366	0.001	2.100 ± 0.876	1.000 ± 0.707	0.04	2.091 ± 0.831	0.750 ± 0.500	0.001	0.074	0.794

Data are shown as mean ± SD; Con: control group, RA: RA group, act: active group, Sta: stable group, Sy: symptomatic group, Asy: asymptomatic group, Dur: duration. The *P* value was calculated by using the Mann–Whitney *U* test or the spearman correlation test. *R*: coefficient of spearman correlation test.

## Data Availability

The detailed data of individual cases used to support the findings of this study are available from the corresponding author upon request.
